# Expression of RNA virus proteins by RNA polymerase II dependent expression plasmids is hindered at multiple steps

**DOI:** 10.1186/1743-422X-4-51

**Published:** 2007-06-05

**Authors:** Nicola Ternette, Daniela Stefanou, Seraphin Kuate, Klaus Überla, Thomas Grunwald

**Affiliations:** 1 Department of Molecular and Medical Virology, Ruhr-Universität Bochum, 44780 Bochum, Germany

## Abstract

**Background:**

Proteins of human and animal viruses are frequently expressed from RNA polymerase II dependent expression cassettes to study protein function and to develop gene-based vaccines. Initial attempts to express the G protein of vesicular stomatitis virus (VSV) and the F protein of respiratory syncytial virus (RSV) by eukaryotic promoters revealed restrictions at several steps of gene expression.

**Results:**

Insertion of an intron flanked by exonic sequences 5'-terminal to the open reading frames (ORF) of VSV-G and RSV-F led to detectable cytoplasmic mRNA levels of both genes. While the exonic sequences were sufficient to stabilise the VSV-G mRNA, cytoplasmic mRNA levels of RSV-F were dependent on the presence of a functional intron. Cytoplasmic VSV-G mRNA levels led to readily detectable levels of VSV-G protein, whereas RSV-F protein expression remained undetectable. However, RSV-F expression was observed after mutating two of four consensus sites for polyadenylation present in the RSV-F ORF. Expression levels could be further enhanced by codon optimisation.

**Conclusion:**

Insufficient cytoplasmic mRNA levels and premature polyadenylation prevent expression of RSV-F by RNA polymerase II dependent expression plasmids. Since RSV replicates in the cytoplasm, the presence of premature polyadenylation sites and elements leading to nuclear instability should not interfere with RSV-F expression during virus replication. The molecular mechanisms responsible for the destabilisation of the RSV-F and VSV-G mRNAs and the different requirements for their rescue by insertion of an intron remain to be defined.

## Background

Eukaryotic cells differ from prokaryotic cells by increased compartmentalisation of the intracellular environment to facilitate complex enzymatic reactions required for efficient protein expression and modification, cell metabolism and/or cell division. Adaptation to the host cell and particularly to its expression machinery is the key requirement for the replication of any virus. Several RNA viruses only replicate in the cytoplasm of their eukaryotic host cell. These viruses possess their own transcription machinery involving a viral RNA-dependent RNA polymerase which allows cytoplasmic mRNA synthesis from the viral genomic RNA. Therefore, these viruses are not adapted to the complex nuclear milieu of the eukaryotic host cell. Inefficient expression of genes from RNA viruses by RNA polymerase II (Pol II) dependent cellular promoters might be explained by lack of critical elements required for pre-mRNA stabilisation, mRNA processing and/or nuclear export. However, problems that occur during Pol II dependent expression of RNA virus proteins can be overcome by changing the codons of viral genes to those most frequently used by the genes of the host cells [1-3]. Since the codon optimised genes should also lack defined RNA elements directing mRNA processing and/or transport, the nucleotide sequence or composition of the viral wild type sequences might actually be inhibitory in nature or be targeted by innate viral defence mechanisms.

The precise reason why genes of RNA viruses are inefficiently expressed is still poorly understood. For lentiviruses, which were studied in more detail, expression of viral structural genes is regulated at the level of nuclear export and these viruses have a regulatory protein (Rev) involved in shuttling the mRNA for the structural proteins from the nucleus to the cytoplasm [4]. Retention of these lentiviral mRNAs in the nucleus has been attributed to *cis*-repressive sequences or regions of instability but these sequences could not be narrowed down to well-defined nucleotide motifs. The unusual low GC content has also been reported to be responsible for the nuclear instability of lentiviral structural mRNAs [5]. Whether similar mechanisms govern the fate of recombinant Pol II mRNAs of viruses replicating in the cytoplasm is unclear.

Instead of using cellular RNA polymerases for expression of viral proteins in eukaryotic cells, cytoplasmic expression systems based on RNA polymerases from vaccinia viruses, alpha-viruses or phages have been developed. The latter are also used for generation of recombinant vesicular stomatitis virus (VSV) [6,7] and respiratory syncytial virus (RSV) [8] by reverse genetics. These systems are based on cytoplasmic transcription of viral cDNA by coexpression of phage T7 RNA polymerase. Recovery of infectious viruses was achieved by cotransfection of T7 RNA polymerase dependent expression plasmids for full-length antigenomic RNA and viral helper proteins which are necessary and sufficient for both RNA-replication and transcription. Expression of these viral helper proteins and/or the antigenomic RNA transcripts by eukaryotic promoters might facilitate and improve strategies for production of such recombinant viruses.

Additionally, the lack of eukaryotic expression systems not depending on coexpressed cytoplasmic polymerases hampered DNA vaccine development for several RNA viruses. This is a particular problem for the development of RSV vaccines, since immunisation with whole inactivated virus particles led to enhancement of RSV disease in children not protected from RSV infection [9,10]. An aberrant T-helper cell type 2 response to the G protein of RSV and excessive CD4+ and CD8+ T cell responses to the F protein of RSV might be responsible for the enhanced airway inflammation underlying the detrimental effect of vaccination [11]. Expression of a single viral protein by a DNA vaccine triggering T-helper cell type 1 responses might overcome vaccine-induced enhancement of RSV disease.

The potential of DNA vaccines and techniques used for reverse genetics has sparked our interest to better understand the requirements for expression of heterologous genes not adapted to the nuclear environment. Using the open reading frames of the G protein of VSV and the F protein of RSV as representatives of the rhabdovirus and paramyxovirus family, respectively, we analysed expression efficiency on mRNA and protein levels. We also attempted to rescue expression of these viral ORF by more subtle changes than codon optimisation to get hints on mechanisms responsible for inefficient expression of these viral genes.

## Results

### Expression of the VSV-G protein can be rescued by insertion of the CMV-IE 5'-untranslated region independent of splicing

**Figure 1:**
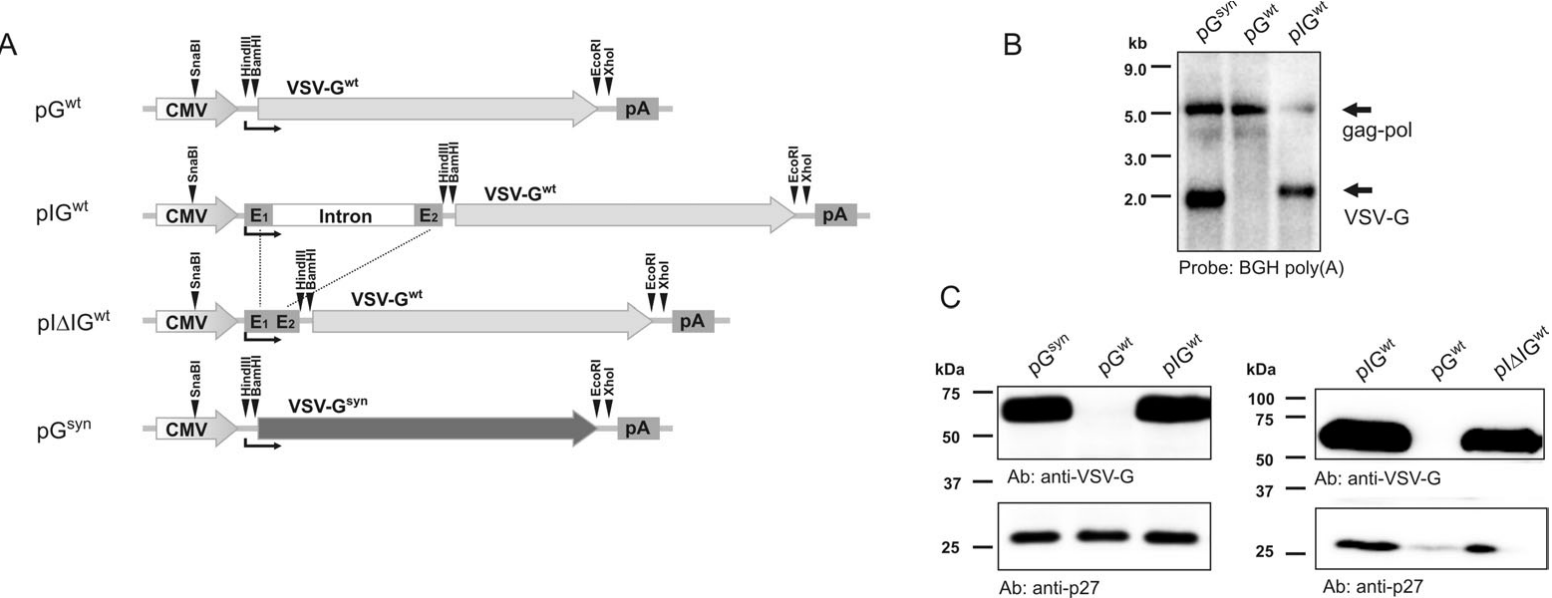
**Characterisation of VSV-G expression plasmids**. A) Map of VSV-G expression plasmids. Wild type (wt) or codon optimised (syn) open reading frames of VSV-G are flanked by the human cytomegalovirus immediate early promoter/enhancer region (CMV) and the bovine growth hormone poly(A) signal (pA). Angled black arrows mark the transcriptional start point. The pIG^wt ^vector contains intron A and flanking untranslated exonic regions E1 and E2 of the cytomegalovirus immediate early gene. In pIΔIG^wt ^the exon boundaries were precisely fused by deleting the intron. B) Northern blot analysis. Cells were cotransfected with the indicated VSV-G expression plasmids, a codon optimised HIV-1 *gag-pol *expression plasmid (Hgp^syn^) and the lentiviral vector construct VICGΔBH containing a GFP expression cassette. Poly(A) RNA was isolated from transfected cells and analysed by Northern blot with a probe spanning the transcribed region of the BGH poly(A) signal present on all VSV-G transcripts and the positive 5 kb HIV-1 *gag-pol *transcript. C) Western blot analyses. Cells were cotransfected with the indicated VSV-G expression plasmids, an SIV *gag-pol *expression plasmid (SgpΔ2) and the lentiviral vector construct VICGΔBH containing a GFP expression cassette. Monoclonal antibodies to HIV-1 p24 capsid protein, which is cross reactive to SIV p27, or to VSV-G, respectively, were used for detection of the viral proteins in lysates of transfected cells.

Heterologous genes are commonly expressed in eukaryotic cells by cloning the ORF into a Pol II dependent expression vector containing a strong constitutive promoter and a polyadenylation signal (poly(A) signal). For expression of the G protein of VSV expression plasmids pG^wt ^and pG^syn^, containing either the wild type or codon optimised ORF under the control of the human cytomegalovirus immediate early promoter and enhancer (CMV-IE, [12]), were transfected into 293T cells (Fig. 1A). Neither mRNA nor protein expression could be detected after transfection of the wild type constructs (Fig. 1B, C). By contrast, the codon optimised construct led to efficient expression of both VSV-G mRNA and protein. Since the probe used for the Northern blot analysis targets the transcribed region of the bovine growth hormone polyadenylation signal (BGH poly(A) [13]) present in the codon optimised and the wild type expression plasmid, the intensity of the bands should directly reflect mRNA expression levels. Since the amino acid sequences encoded by the codon optimised and the wild type ORF are identical, both proteins should be detected with the same sensitivity in Western blot analysis. Detection of VSV-G after transfection of the codon optimised expression plasmid also excludes the possibility that lack of detectable levels of VSV-G after transfection of the wild type construct is due to instability of the protein. The VSV-G expression plasmids were cotransfected with lentiviral *gag-pol *expression plasmids and a lentiviral vector construct to assess expression levels of VSV-G by a sensitive VSV-G dependent transduction assay. Vector titers obtained with the wild type construct were at least 100-fold lower than those obtained with the codon optimised expression plasmid (data not shown). Since the cotransfected *gag-pol *expression plasmid also contained the BGH poly(A), the Northern blot analyses also detected the encoded *gag-pol *mRNA migrating at a size of approximately 5 kb. Similar *gag-pol *mRNA levels (Fig. 1B) confirm that the differences observed in VSV-G expression are not due to experimental variations.

Inserting the first intron of CMV-IE gene including exonic flanking regions restored VSV-G expression from the wild type ORF to levels comparable to those obtained by the codon optimised expression plasmid. Despite a lower transfection efficiency, as evident from the Northern blot analysis (Fig. 1B), VSV-G mRNA expression was clearly detectable (pIG^wt ^in Fig. 1B). Protein expression levels were comparable to those obtained with the codon optimised expression plasmid (pIG^wt ^vs pG^syn ^in Fig. 1C, left panel). However, splicing was not required for this rescue, since a DNA expression plasmid, in which the CMV intron had been deleted by fusing the splice sites and retaining the exonic sequences also led to efficient expression of the protein (compare pIG^wt ^to pIΔIG^wt ^in Fig. 1C, right panel). Thus, correctly fused exons were sufficient to enhance VSV-G expression levels.

Further deletion analyses revealed that the first 106 nucleotides of the 5'-exon are mediating most of the effect (data not shown).

### Expression of the RSV-F mRNA is dependent on splicing

**Figure 2:**
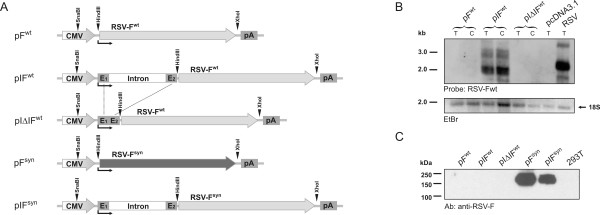
**Characterisation of RSV-F expression plasmids**. A) Map of RSV-F expression plasmids. Wild type (wt) or codon optimised (syn) open reading frames of RSV-F are flanked by the human cytomegalovirus immediate early promoter/enhancer region (CMV) and the bovine growth hormone poly(A) signal (pA). Angled black arrows mark the transcriptional start point. The pIF^wt ^and pIF^syn ^plasmids contain intron A and flanking untranslated exonic regions E1 and E2 of the cytomegalovirus immediate early gene. In pIΔIF^wt ^the exon boundaries were precisely fused by deleting the intron. B) Northern blot analysis. 293T cells were transfected with the indicated plasmids containing the RSV-F ORF. As a negative control the empty vector (pcDNA3.1) was also transfected and processed in parallel. A total RNA extract from RSV-infected HEp2-cells served as a positive control. Cytoplasmic (C) or total (T) RNA was isolated from transfected cells, separated by agarose gel electrophoresis and used for subsequent Northern blot analysis. Size separated RNA was stained with ethidiumbromide (EtBr) revealing non-degraded 18S and 28S ribosomal RNA bands (18S shown as representative). A DIG-labelled probe spanning 780 bp of the RSV-F ORF was used for hybridisation. C) Western blot analysis. 293T cells were transfected with the indicated plasmids. Equal amounts of protein were separated on an acrylamide gel for subsequent detection of RSV-F expression in Western blot analysis using a monoclonal antibody against the F protein. As a positive control, RSV-infected HEp2 cells were processed in parallel.

An analogous expression plasmid containing the wild type RSV-F ORF under the control of the CMV-IE promoter-enhancer (pF^wt ^in Fig. 2A) also failed to express detectable levels of RSV-F mRNA and protein (Fig. 2B,C). After insertion of the first intron of CMV-IE gene with the exonic flanking regions into the wild type RSV-F expression plasmid, RSV-F mRNA could be detected in the cytoplasm of transfected cells (pIF^wt ^in Fig. 2B). However, RSV-F protein remained undetectable. In contrast to VSV-G, splicing seemed to be required, as selective removal of the intronic sequences (pIΔIF^wt^) abolished detection of cytoplasmic RSV-F mRNA (Fig. 2B). To exclude the possibility that our failure to detect RSV-F protein is due to instability of RSV-F or poor antibody reagents, we also analysed a codon optimised RSV-F expression plasmid encoding the same RSV-F amino acid sequence as the wild type constructs. Expression of RSV-F was readily detectable after transfection of the codon optimised expression plasmid independent of the presence or absence of the intron (pF^syn ^and pIF^syn ^in Fig. 2C).

### Pol II mediated expression of the wild type RSV-F ORF results in premature polyadenylation

Undetectable levels of RSV-F protein in the presence of cytoplasmic RSV-F mRNA suggested an additional block at the translational level. We noticed that the mRNA species detected in the Northern blot analysis (Fig. 2B) migrated faster than the viral RSV-F mRNA, although they should be slightly larger due to the extended 5'- and 3'-UTR.

**Figure 3:**
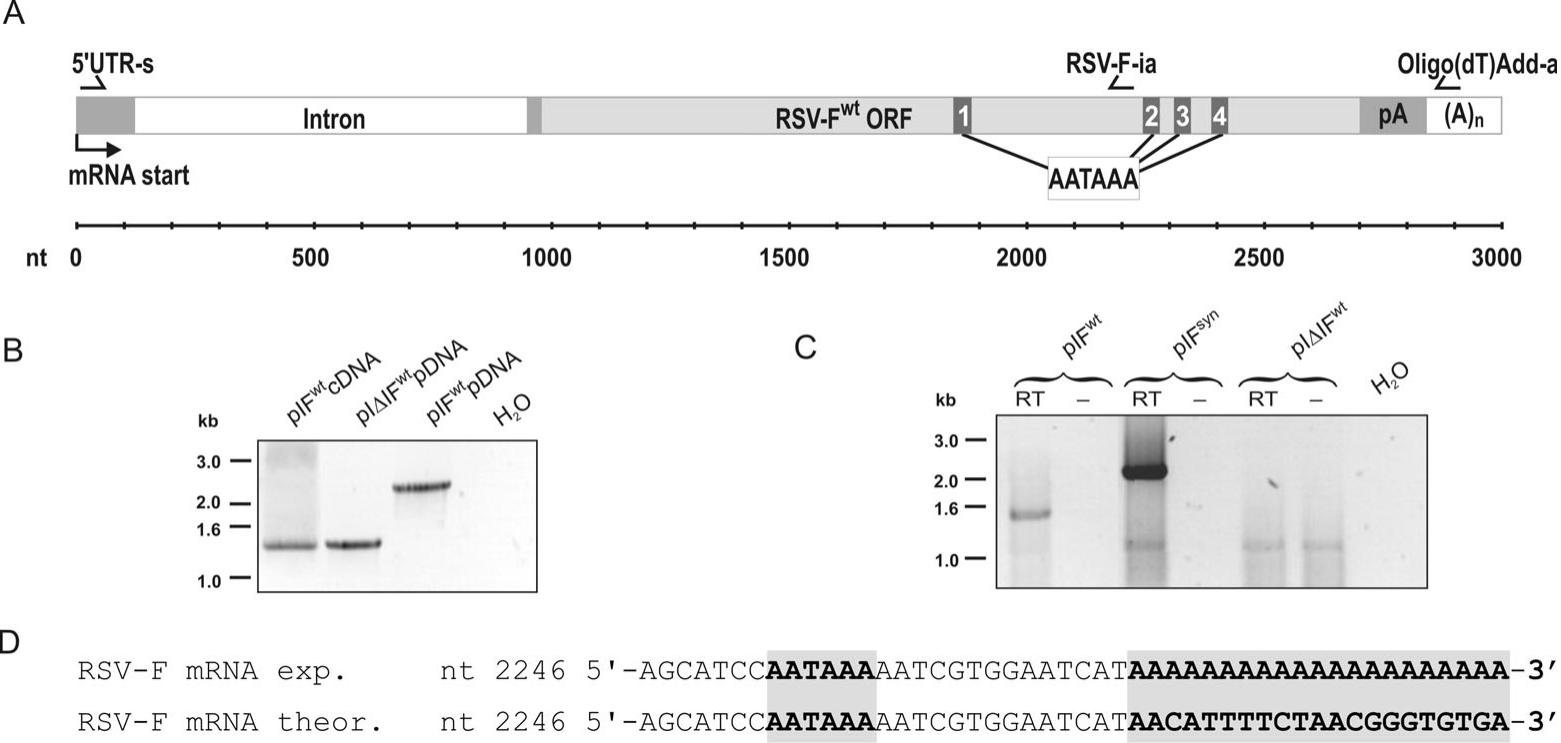
**Analysis of RSV-F mRNA processing**. A) Map of exon-intron structure and poly(A) signals of the precursor mRNA encoded by pIF^wt^. Arrows indicate location of primers used for the PCR analyses. The scale indicates the distance to the transcriptional start site. AATAAA: consensus signal for polyadenylation. B) Characterisation of splicing. 293T cells were transfected with pIF^wt^. Cytoplasmic RNA was isolated from transfected cells and reverse transcribed by oligo-dT priming (pIF^wt^cDNA). A PCR spanning the splice sites was performed with primers: 5'UTR-s and RSV-F-ia. The size of the PCR-products was compared to the size obtained in parallel PCR using pIΔIF^wt ^and pIF^wt ^plasmid-DNA (pDNA) as templates. H_2_O: negative control. C) Characterisation of poly(A) signal usage. 293T cells were transfected with the indicated plasmids. Cytoplasmic RNA was isolated from transfected cells and reverse transcribed by priming with Oligo(dT)Add-a. As a control for DNA contamination, the reverse transcription reaction was also performed without the enzyme (-). The cDNA was amplified by PCR with primers 5' UTR-s and Oligo(dT)Add-a and the size of the PCR products was determined by agarose gel electrophoresis. D) PCR products from pIF^wt ^transfected cells were cloned and sequenced. The 3' end of the sequence obtained in 9 of 10 clones (RSV-F mRNA exp.) is shown aligned to the RSV-F sequence of the parental plasmid (RSV-F mRNA theor.).

To analyse correct splicing of the RSV-F mRNA, cytoplasmic RNA of 293T cells transfected with pIF^wt ^was isolated and reverse transcribed by an oligo-dT primer. A PCR spanning the splice sites in the 5'-untranslated region was performed (see Fig. 3A). Size and sequence analysis (not shown) of the PCR product revealed correct splice junctions and no other deviation from the expected transcript (Fig. 3B).

However, inspection of the RSV-F sequence revealed four potential polyadenylation consensus signals (AATAAA) [14] within the coding region (Fig. 3A). Using a PCR approach with an antisense primer anchored at the poly(A) tail (Oligo(dT)Add-a, Fig. 3A) and a pcDNA3.1+ specific sense primer at the 5'-UTR of the transcript (5' UTR-s, Fig. 3A), the entire mRNA transcript was reverse transcribed and amplified. Size and sequence analysis revealed that the second consensus poly(A) signal was used in 9 of 10 clones analysed (Fig. 3C, D) resulting in a mRNA with an RSV-F ORF truncated at position 1295. Since the absence of a stop codon has been shown to lead to degradation of such prematurely terminated proteins by cellular quality control pathways [15,16], this might explain the absence of detectable levels of a truncated protein.

### Deletion of the poly(A) consensus signal rescues RSV-F expression

**Figure 4:**
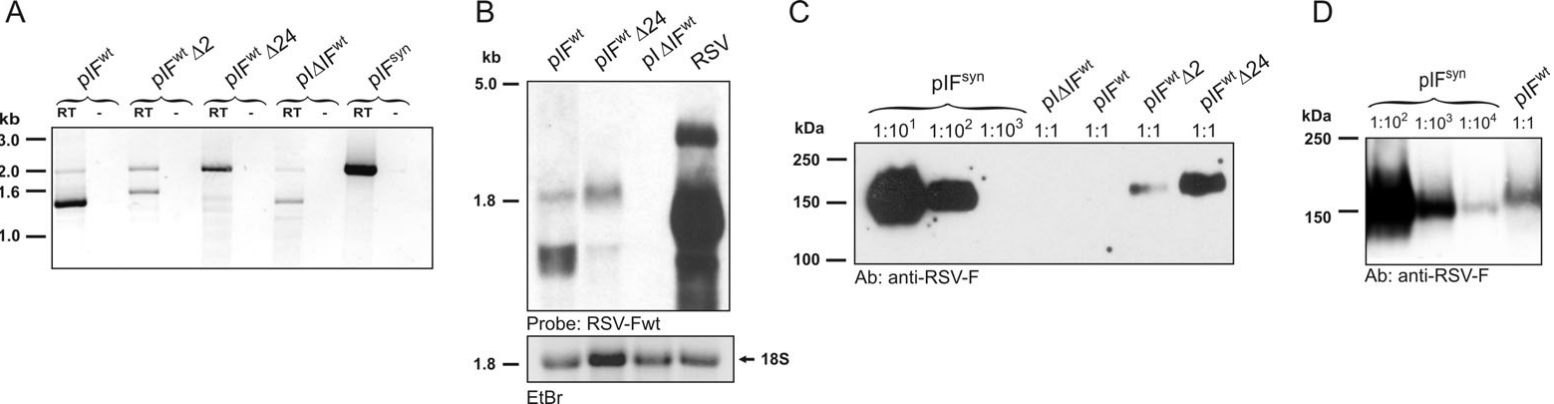
**Characterisation of poly(A) signal mutants**. A) Poly(A) signal usage after transfection of the indicated plasmids into 293T cells was characterised as described in figure legend 3C. Plasmids pIF^wt ^Δ2 and pIF^wt ^Δ24 contain mutations in the second or the second and forth consensus poly(A) signal, respectively. B) Northern blot analysis of cytoplasmic RNA of 293T cells transfected with the indicated plasmids or HEp2 cells infected with RSV. Size separated RNA was stained with ethidiumbromide (EtBr) revealing non-degraded 18S and 28S ribosomal RNA bands (18S shown as representative). A DIG-labelled probe from the RSV-F ORF was used for hybridisation. C, D) Western blot analysis. 293T cells were cotransfected with an EGFP expression plasmid and the indicated RSV-F expression plasmids and analysed by Western blot using an anti-RSV-F monoclonal antibody. The lysate of pIF^syn ^transfected cells was diluted from 1:10 to 1:10^4^, while the lysates from the other transfected cell were loaded at a 1:1 dilution. Similar transfection efficiencies were controlled for by measuring the fluorescence activity of cell lysates (data not shown).

Mutation of the consensus poly(A) signal 2 by a point mutation not affecting the protein sequence at position 1278 of the ORF (1^st ^mutation: AATAAA → AACAAA in pIF^wt^Δ2) led to detectable full-length transcripts (Fig. 4A) and a faint RSV-F band became detectable in the Western blot analysis (Fig. 4C). Despite detection of full length transcripts, RSV-F protein levels after transfection of pIF^wt ^Δ2 were more than 100-fold lower than those obtained after transfection of the codon optimised expression plasmid. In addition to full length transcripts a second band consistent in size with polyadenylation at the fourth consensus poly(A) signal was obtained by PCR analysis of RSV-F transcripts (Fig. 4A), which might be responsible for the poor expression observed at the protein level. Therefore, the second and fourth poly(A) signal were inactivated simultaneously by introducing an additional silent point mutation at position 1425 of the RSV-F ORF (2^nd ^mutation: AATAAA → AATCAA: pIF^wt^Δ24). This led to detection of only full length mRNA transcripts (Fig. 4A, B) and expression of RSV-F protein was increased relative to the pIF^wt ^Δ2 construct. However, despite detection of full-length mRNA in the cytoplasm, protein expression was at least 50-fold less efficient than expression from the codon optimised plasmid (Fig. 4C).

### Virus infection does not enhance Pol II dependent RSV-F expression

**Figure 5:**
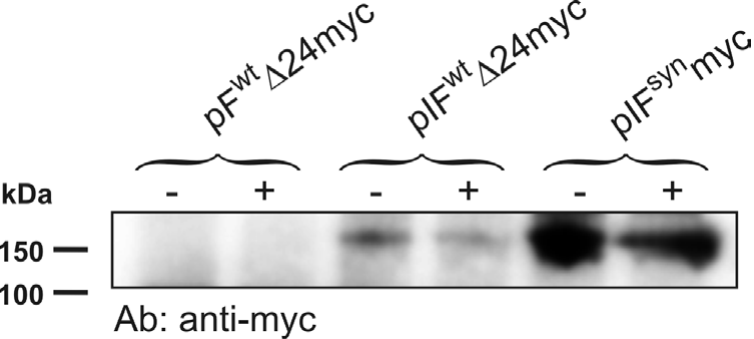
**Influence of RSV infection on RNA Pol II dependent RSV-F expression levels**. 293T cells were cotransfected with a *Gaussia *luciferase expression plasmid and the indicated RSV-F expression plasmids containing an inframe 3'-terminal myc-tag. Similar transfection efficiencies were controlled by measuring the *Gaussia *luciferase activity in cell supernatants (not shown). Six hours following transfection cells were infected with the GFP expressing recombinant RSV at an MOI of 2 (+). As negative control, cells were also left uninfected (-). GFP expression analysis revealed similar infection efficiencies (data not shown). Equal amounts of protein were analysed in non-reducing Western blot analysis using a monoclonal antibody to the myc-tag 48 h after transfection.

Since the wild type ORF of RSV-F is readily translated in the context of the virus, this inefficient expression does not seem to be a general deficiency of the translation machinery or a consequence of rare codon usage. Due to the cytotoxicity of RSV-F we hypothesised that unidentified *cis*-repressive sequences in the wild type ORF might participate in regulation of RSV-F protein expression during the viral replication cycle, and that another viral protein might activate RSV-F protein expression at the translational level. To test this possibility, cells transfected with the wild type RSV-F expression plasmid with inactivated premature poly(A) signals were infected with RSV. To distinguish between RSV-F protein expressed from the expression plasmid or the virus, a 10 amino acid myc-tag was added to the C-terminus of the RSV-F ORF in pIF^wt^Δ24 resulting in pIF^wt^Δ24myc. Recombinant RSV expressing GFP was used and infection efficiency was controlled by fluorescence microscopy. Expression levels of myc-tagged RSV-F in transfected cells were not enhanced by viral infection (Fig. 5), thus failing to provide evidence for upregulation of RSV-F expression by another viral factor.

### Chimeric ORF of RSV-F revealed strong dependency of protein expression on codon usage

**Figure 6:**
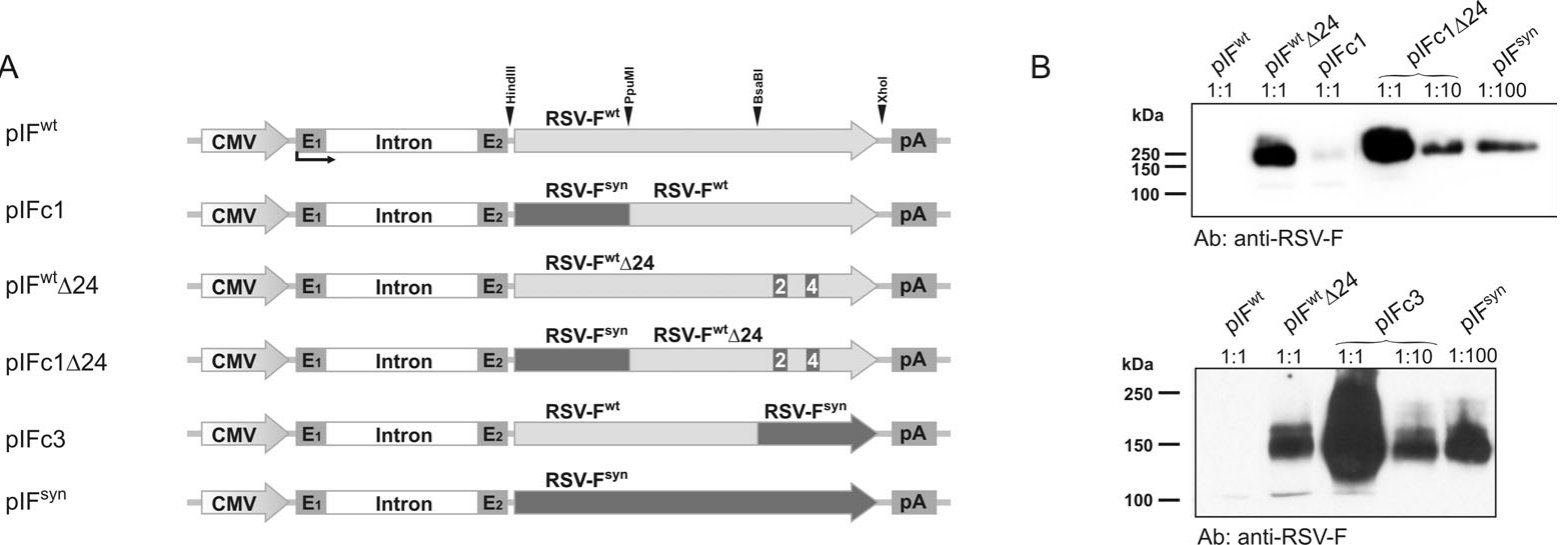
**Expression levels of chimeric ORFs**. A) Map of RSV-F expression plasmids containing wild type, codon optimised, or chimeric ORFs. Numbers in boxes indicate mutated consensus poly(A) signals. B) Western blot analysis of RSV-F protein levels after transfection of the indicated expression plasmids. An expression plasmid for EGFP was cotransfected. Undiluted lysates had equal protein content and a similar amount of fluorescent activity revealing constant transfection efficiency. Numbers above the lanes indicate the dilution at which the cell lysates were loaded on the gel.

To dissect the functional relevance of codon optimisation and premature polyadenylation more precisely, we also replaced the first 466 nt (pIFc1) or last 679 nt (pIFc3) of the wild type sequence with the codon optimised form (Fig. 6A). In the presence of functionally active premature poly(A) signals (pIFc1) replacement of the first third of the wild type ORF by the codon optimised version did not restore protein expression. Increased expression levels were obtained with the chimeric construct in which the relevant poly(A) signals were deleted (pIFc1Δ24) relative to the wild type sequence containing the mutated polyadenylation sites (pIF^wt ^Δ24). A comparable increase of expression was observed, if the last third of the wild type ORF was exchanged by the codon optimised sequence. Codon optimisation of the entire sequence even led to at least 10-fold higher expression levels compared to the chimeric constructs, indicating that codon optimisation seems to be the major reason for the strong enhancement of protein levels once premature poly(A) signals are inactivated.

## Discussion

The results demonstrate striking differences in the requirements for expression of genes of cytoplasmic RNA viruses by DNA expression plasmids. The use of codon optimised expression plasmids allowed exclusion of the possibility that protein instabilities or degradation is responsible for undetectable levels of the respective viral proteins. Insertion of intron A of the CMV-IE gene resulted in mRNA levels comparable to those obtained by codon optimised expression plasmids in case of VSV-G or by those obtained in natural infection for RSV. This was not surprising since splicing has been repeatedly shown to enhance expression levels [17,18]. However, in case of VSV-G splicing was not the critical factor, since simple addition of the exonic sequences of the CMV-IE gene were sufficient to rescue VSV-G expression even at the protein level. This suggests that the exonic sequences somehow stabilise and/or contribute to nuclear export of the VSV-G mRNA. The same exonic sequences were not sufficient to rescue expression of RSV-F mRNA. Including the intron, however, allowed cytoplasmic expression of the RSV-F mRNA. Similar findings have been reported for mRNAs of the Simian Virus 40, where intronless RNA was retained and degraded in the nucleus, while the same transcript generated by splicing reached the cytoplasm [19]. For other genes this has been attributed to recognition of the pre-mRNA by the exon junction complex (EJC), which has been found to be linked to nuclear export by direct binding to the heterodimer transport protein Tap-Nxt [20-22]. It is therefore likely that similar mechanisms are responsible for the rescue of cytoplasmic RSV-F mRNA levels by the first intron of the CMV-IE gene.

Another block to RSV-F protein synthesis was found to be premature polyadenylation. The second of the four consensus sites initiated the predominant premature polyadenylation of the RSV-F mRNA. The lack of a stop codon preventing an accurate translation termination results in synthesis of defective ribosomal products (DRiPs) which enter a pathway of proteasomal or other cytosolic decay mechanisms coupled to MHC class I presentation [23,24]. This might explain why DNA vaccines encoding the wild type RSV-F ORF induced immune responses, although expression of full length protein was probably not very efficient [25-29]. The small amount of protein which could be detected despite that (Fig. 4D), might be the result of a rare skipping of poly(A) signals.

Mutagenesis of the recognised consensus sequence for polyadenylation led to usage of the last downstream consensus signal. However, even after mutagenising both used poly(A) signals, protein expression was around 50-fold lower than expression from codon optimised plasmids despite substantial amounts of correctly processed RSV-F mRNA in the cytoplasm. The fact that expression of other viral proteins by superinfection of the transfected cell with RSV did not increase RSV-F expression from the plasmid suggests that poor expression levels are not the consequence of a repressive RSV-F specific regulatory RNA element that can be overcome by an activating second viral factor. It rather seems that reducing the AU content of the RSV-F mRNA in general contributes to increased expression levels. Consistently, replacement of a third of the wild type nucleotide sequence by the codon optimised fragment resulted in intermediate expression levels and not in an all or none phenomenon. In summary, these findings indicate that premature polyadenylation is the major mechanism responsible for failure of protein expression from the original RSV-F wild type construct and that codon optimisation can further enhance expression of RSV-F.

Polyadenylation consensus signals were not only found in a single RSV strain but could be detected in all RSV-F sequences deposited in GenBank database. Other members of the paramyxovirus family, such as measles virus and parainfluenzaviruses, also harbour such consensus sites for polyadenylation. Since these consensus poly(A) signals are not expected to be of any functional relevance for the viruses due to their cytoplasmic replication, they are probably just the accidental result of the unusual high AU content of the viral genomes. The latter fact also leads to the presence of potential U-rich downstream elements that are also required for polyadenylation [30,31].

## Conclusion

Expression of genes of RNA viruses by Pol II dependent expression plasmids can be impaired at several steps. For VSV-G, a splicing-independent mechanism can lead to stabilisation of Pol II transcribed VSV-G mRNA, while splicing seems to be necessary for Pol II dependent expression of RSV-F mRNA. Premature polyadenylation is a second major block to expression of RSV-F protein from the wild type ORF. All these restrictions were efficiently overcome by codon optimisation providing a straightforward approach for the generation of Pol II dependent expression cassettes needed for development and production of antiviral vaccines and recombinant RNA viruses.

## Methods

### Viruses and infection

RSV based on the A2 long strain was kindly provided by B. Schweiger from the Robert Koch Institute, Berlin, Germany. GFP expressing recombinant rgRSV [32] was obtained by M. E. Peeples and P. L. Collins, Maryland, USA. RSV was passaged on Hep2 cells and stored at -80°C. Hep2 or 293T cells were infected at an MOI of 10 by adding RSV containing cell supernatant. Two hours following addition of the virus, supernatants were removed and cells were supplied with DMEM medium containing 0,5% FCS and 100 μg/ml penicillin G and streptomycin sulphate.

### Expression plasmids

The ORF of VSV-G was amplified from pHIT-G [33] and cloned into pcDNA3.1 (Invitrogen, Karlsruhe, Germany) via BamHI/EcoRI (pG^wt^, kindly provided by R. Wagner, Regensburg). A Kozak consensus sequence (gccgccacc) [34] was inserted directly upstream of the start codon. For codon optimisation, viral codons were replaced by those most frequently used in human cells [35]. Synthesis of the optimised VSV-G encoding nucleotide sequence was performed by Geneart (Regensburg, Germany) based on the amino acid sequence of GenBank database entry J02428 (pG^syn^). Amino acid 57 and 96 were mutated from L to I and H to Q, respectively, to match the amino acid sequence of the wild type VSV-G precisely. The CMV-IE intron A was added into both vectors by inserting the SnaBI/HindIII fragment (GenBank database entry BK000394, nt 174903–173696) of the VSV-G expression plasmid pHIT-G resulting in pIG^wt ^and pIG^syn^.

Deletion of the 828 nt intron and exact fusion of the exon boundaries was achieved by replacement of a SacII/HindIII fragment by the annealed oligonucleotides (Sigma, Munich, Germany) Is (5'-**gg**ccgggaacggtgcattggaacgcggattccccgtgccaagagtgactcaccgtccttgacacg**a**) and Ia (5'-**agctt**cgtgtcaaggacggtgagtcactcttggcacggggaatccgcgttccaatgcaccgttcccgg**ccgc**) resulting in pIΔIG^wt^. Nucleotides involved in generation of restriction sites are printed bold. VSV-G expression analyses included studies on the functional incorporation of VSV-G into lentiviral vector particles. Therefore, lentiviral *gag-pol *expression plasmids Hgp^syn ^[2] for HIV-1 *gag-pol *and SgpΔ2 [36] for SIV *gag-pol *were cotransfected with VSV-G expression plasmids and the lentiviral vector construct VICGΔBH. VICGΔBH is based on the lentiviral vector VIGΔBH [36], containing a murine leukemia virus promoter driven GFP expression cassette. This cassette was excised via BglII/XhoI and replaced by the BamHI/XhoI fragment of HIV-CS-CG [37] containing a CMV-GFP expression cassette.

For the construction of the RSV-F expression plasmids, viral RNA was isolated from RSV containing cell supernatants using the QIAamp^® ^viral RNA Mini Kit. After reverse transcription (ThermoScript™ RT-PCR System, Invitrogen) the RSV-F cDNA was amplified by PCR (Primers (Sigma): sense: 5'-gatcc**aagctt**ccaccatggagttgccaatcctcaaa; antisense: 5'-tcgac**ctcgag**ttagttactaaatgcaatattatttatacc) using the Platinum^® ^Taq DNA-polymerase (Invitrogen). The 1.7 kb fragment including a Kozak sequence upstream of the ORF (ccacc) was subcloned into pcDNA3.1 (Invitrogen, Karlsruhe, Germany), or used to replace the VSV-G sequence pIG^wt ^and pIΔI by digestion with HindIII/XhoI. Codon optimisation of the wild type ORF was performed by Geneart. The codon optimised ORF (GenBank database entry EF566942), also including a Kozak sequence (gccacc), was subcloned into pcDNA3.1 (Invitrogen) and pI vector by HindIII/XhoI restriction.

Deletion of the stop codon of the RSV-F ORF was achieved by PCR-directed mutagenesis. The RSV-F^syn ^ORF without the stop codon was then subcloned into the pcDNA3.1(+) vector and the myc-tag was fused to the C-terminus of RSV-F by ligating annealed primers (Sigma) mycTAAs: 5'-**tcgag**gaacaaaaactcatctcagaagaggatctgtaat and mycTAAa: 5'-**ctaga**ttacagatcctcttctgagatgagtttttgttc**c **into the expression plasmid containing the RSV-F ORF lacking the stop codon via XhoI and XbaI sites.

Point mutations were introduced to the RSV-F ORF by overlap extension PCR and ligation of PpuMI/XhoI fragments.

Chimeric ORFs were produced by amplification of portions of the synthetic ORF and subcloning via HindIII/PpuMI or BsaBI/XhoI into the wild type expression vector pIF^wt^. All plasmids were confirmed by sequence analysis (Genterprise, Mainz, Germany).

### Cells and transfection

293T and HEp2 cells were cultured in Dulbecco s modified Eagle s medium (Invitrogen) supplemented with 10% fetal calf serum (Invitrogen), penicillin G and streptomycin sulphate in a final concentration of 100 μg/ml each. Cells were transfected in 25 cm^2 ^flasks with 5 μg plasmid-DNA by the calcium phosphate coprecipitation method as described elsewhere [38].

### Control of transfection efficiency

To control transfection levels and guarantee comparable amounts of protein in lysates of transfected cells, plasmids for expression of reporter proteins were transfected additionally to VSV-G and RSV-F expression plasmids. In case of VSV-G expression analyses, cotransfection of lentiviral *gag-pol *expression plasmids Hgp^syn ^[2] for HIV-1 *gag-pol *and SgpΔ2 [36] for SIV *gag-pol *served as control in Western and Northern blot analyses, respectively. Cotransfection of the lentiviral vector VICG3ΔBH containing a GFP-expression cassette directly monitored transfection efficiency in treated cells. For RSV-F expression analyses cotransfection of an EGFP expression plasmid (pEGFP-C1, BD Biosciences Clontech, Heidelberg, Germany) and quantitative measurement of fluorescence activity in cell lysates guaranteed similar transfection efficiency. In transfected cells subsequently infected with rgRSV, transfection efficiency was controlled by transfection of an expression plasmid for Gaussia luciferase (pCMV-GLuc1; Targeting Systems, Santee, USA) and measurement of its activity in cell supernatants.

### Western blot analysis

Transfected 293T cells were lysed 48 h following transfection. Equal amounts of total protein measured by Bradford-Assay (Biorad, Munich, Germany) were loaded on sodium dodecyl sulphate 8–12% polyacrylamide gels in reducing (500 mM TrisHCl pH 6,8; SDS; 20 v/v β-mercaptoethanol; 40 v/v Glycerin; 0,04% (w/v) PyroninY) or non reducing (without β-mercaptoethanol) Laemmli buffer. After protein separation and blotting on nitrocellulose membrane, proteins were incubated at 4°C over night with monoclonal antibody against either VSV-G (P5D4, Sigma-Aldrich, Munich, Germany), HIV-p24 (AIDS Research and Reference Reagent Program, Dr. Jonathan Allan [39]), RSV-F (18F12 [40]) or the myc-tag (9E10 [41]). After washing, the membrane was incubated with horseradish peroxydase-linked goat-anti-mouse-F_c _antibody (SantaCruz, Heidelberg, Germany) and detected proteins were visualised by enhanced chemiluminescence reaction (Chemiglow^®^, Biozym, Hamburg, Germany).

### Northern blot analysis

Total or cytoplasmic RNA was isolated from transfected 293T cells by RNeasy^® ^Mini Kit (Qiagen, Hilden, Germany), mRNA was isolated by Fast Track 2.0 kit (Invitrogen, Karlsruhe, Germany). Concentration of purified RNA was determined by measuring absorbance at 260 nm. Five μg RNA was separated on an 1% agarose gel and blotted on nylon membrane. DIG-labelled probes where synthesised by PCR using the DIG synthesis kit (Roche, Mannheim, Germany). Transcripts were detected by hybridisation to a probe directed to either the BGH-poly(A) signal of the pcDNA3.1(+) (length: 130 bp; Primers: BGHs: 5'-gagtctagagggcccgtttaa; BGHa: 5'-aggaaaggacagtgggagtg) or the RSV-F ORF (length: 780 bp bp; Primers: RSV-Fis: 5'-ggtcctgcacttagaaggag; RSV-Fia: 5'-catgacacaatggctcctag). Oligonucleotides for probe synthesis PCR were derived from Sigma.

DIG-labelled nucleic acids were visualised by an alkaline phosphatase coupled anti-DIG antibody and CSPD substrate (Roche).

## Competing interests

KÜ and TG have filed a patent application on the use of the codon optimised RSV-F gene.

## Authors' contributions

Cloning of RSV-F expression plasmids and RSV-F expression studies were performed by NT. DS analysed expression of VSV-G and synthesised required plasmids. SK supervised and participated in VSV-G experimental setups. TG and KÜ supervised and attributed to study design and planning. KÜ and TG revised the manuscript written by NT. All authors read and approved the final manuscript.
